# Identifying Household Level Risk Factors for Unintentional House Fire Incidents, Injuries and Fatalities

**DOI:** 10.23889/ijpds.v1i1.335

**Published:** 2017-04-19

**Authors:** Rachael Williams, Arlene Gallagher, Tjeerd van Staa, Tarek Hammad, Bert Leufkens, Frank de Vries

**Affiliations:** 1 1 Clinical Practice Research Datalink; 2 2 Utrecht Institute for Pharmaceutical Sciences, Utrecht University; 3 3 Office of Surveillance and Epidemiology, Food and Drug Administration

## Objective

Electronic health records are increasingly used to investigate associations between antidiabetic therapy and cancer. Misclassification can impact results, especially if differential between comparators. The objective of this study was to estimate cancer misclassification when using primary care or hospital data alone.

## Methods

Adults aged ≥40 years with an insulin or oral antidiabetic prescription in Clinical Practice Research Datalink (CPRD) primary care data at least a year after start of data collection, and no record of type 1 diabetes, were included. Patients were matched by year of birth (stepwise within 5 years), sex and GP practice to up to 1 non-diabetic patient. The cohort was restricted to those eligible for Hospital Episode Statistics (HES) linkage with follow up during the study period (01/04/97-31/12/06). Follow-up started at the maximum of the registration date with the practice, practice up-to-standard date (a CPRD quality metric), and start of study period. Follow-up ended at the minimum of when the patient left the practice, the date CPRD last collected data from the practice, and end of study period. Cancer was identified in CPRD via Read codes and in HES via ICD10 codes. For each cancer case in CPRD, analysis evaluated whether there was a corresponding record in HES coded with same, different or unspecified site. Analysis was repeated for cancers identified in HES.

## Results

53,585 diabetic patients were matched to 47,435 non-diabetic patients. 83% of cancer cases in CPRD had a corresponding record in HES (78% with the same type). Misclassification varied by cancer site, ranging from 3% (stomach cancer) to 57% (nonmelanoma skin cancer). 83% of cancer cases in HES had a corresponding record in CPRD, with all misclassification rates <20%.

## Conclusion

A good level of concordance and low level of misclassification of cancer exist between CPRD primary care data and HES. The value of linking these data for establishing cancer outcomes lies more in the complimentary variables held than in reducing misclassification.

